# Disulfide Bond Mapping of Follitropin Delta, a Recombinant Follicle Stimulating Hormone (rFSH), by X-Ray Crystallography

**DOI:** 10.3390/ph19030380

**Published:** 2026-02-27

**Authors:** Dorin Kalson, Jeremiah S. Joseph, Hila Nudelman, Eyal Kamhi, Shlomo Bakshi

**Affiliations:** 1 Bio-Technology General Ltd., Kiryat Malachi 8310402, Israel; hila.nudelman@btgil.com (H.N.); eyal.kamhi@ferring.com (E.K.); shlomo.bakshi@ferring.com (S.B.); 2 Opilio LLC., Chula Vista, CA 91915, USA; jeremiah.joseph@opilio.us

**Keywords:** rFSH, disulfide bond, crystallography, X-ray

## Abstract

**Background/Objectives:** Follitropin delta is an approved recombinant follicle-stimulating hormone (rFSH) expressed in a human cell line. Correct disulfide connectivity is a critical quality attribute for rFSH, a heterodimeric glycoprotein composed of noncovalently associated α and β subunits and stabilized by an extensive network of intramolecular disulfide bonds. Disulfide characterization is typically performed by mass spectrometry (MS). However, the closely spaced disulfide bonds within the FSH α-subunit are particularly resistant to proteolytic cleavage, complicating conventional MS-based disulfide mapping. **Methods:** To overcome limitations of MS-based methods, an X-ray crystallography strategy was employed using a ternary complex of the recombinant FSH heterodimer with an anti-FSHα Fab and a stabilizing anti-kappa VHH. Crystals of the desialylated rFSH/Fab/VHH complex were obtained and diffraction data were collected. **Results:** The structure of recombinant FSH was determined at 2.29 Å resolution. Electron density surrounding cysteine residues in both the α and β subunits was well defined, allowing unambiguous assignment of all intramolecular disulfide bonds in the crystallized protein. The observed cysteine connectivity is fully consistent with the disulfide architecture of FSH from other sources and supports correct folding of the recombinant Follitropin delta.

## 1. Introduction

Follicle-stimulating hormone (FSH) is a heterodimeric glycoprotein hormone produced by the anterior pituitary gland in response to gonadotropin-releasing hormone (GnRH). FSH functions via the FSH receptor (FSHR), a G-protein-coupled receptor that is expressed mainly in female Granulosa cells (GCs) and male Sertoli cells [1]. In females, FSH stimulates recruitment and growth of antral follicles by binding to its cognate receptor, FSHR, expressed on GC. Upon ligand binding, FSHR stimulates cyclic adenosine-monophosphate (cAMP) mediated signal transduction pathways inducing proliferation, differentiation and function of antral follicles as well as aromatase activity affecting steroidogenesis [2]. FSHR expression on GC increases upon FSH binding and its expression is affected by several other factors including estrogens, fibroblast growth factor, epidermal growth factor, and others. In addition, GC produces inhibins and activins in an FSH dependent manner further affecting FSHR expression, and hence FSH activity using an autocrine mechanism.

Disulfide bridges are covalent linkages formed between the thiol groups of two cysteine residues within a protein. These bonds play a pivotal role in maintaining protein stability and facilitating proper folding [3]. The thiol (-SH) group of cysteine is highly reactive, enabling the formation of disulfide bonds that contribute significantly to the structural integrity of proteins.

These bridges can be found within both the secondary and tertiary levels of protein structure. In the secondary structure, disulfide bonds help stabilize elements such as alpha helices and beta sheets by providing rigid connections that resist unfolding. At the tertiary level, they are essential for preserving the overall three-dimensional conformation of the protein. Disulfide bonds often link cysteine residues located in distant regions of the polypeptide chain, thereby anchoring different parts of the molecule and reinforcing its spatial arrangement [4].

**Figure 1:**
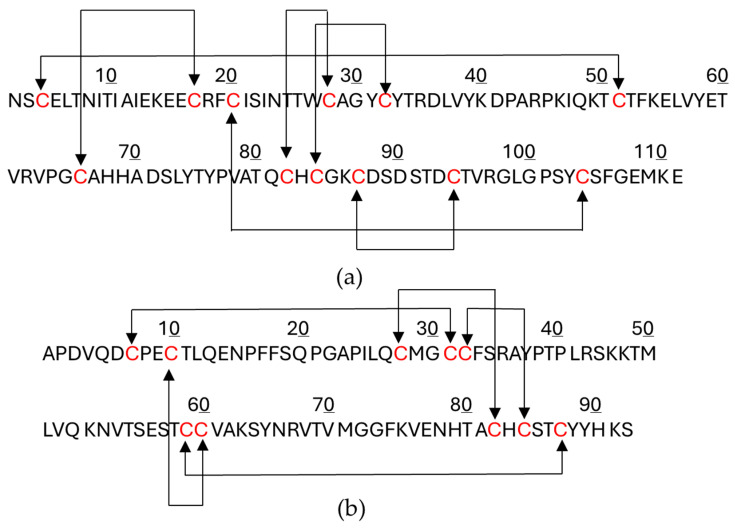
(**a**) The structure of FSH β-subunit (FSHβ) contains six intramolecular disulfide bonds: (C3–C51, C17–C66, C20–C104, C28–C82, C32–C84 and C87–C94). (**b**) The structure of FSH α-subunit (FSHα) contains five intramolecular disulfide bonds: C7–C31, C10–C60, C28–C82, C32–C84 and C59–C87.

FSH is composed of two non-covalently associated subunits, alpha (α) and beta (β). The α—subunit, is common to other gonadotropins, Luteinizing hormone (LH) and Choriogonadotropin (CG), and consists of 92 amino acids. The 111 amino acid β -subunit is specific to FSH and hence mediates specific binding and activation of FSH activity. Both FSH subunits carry N-linked glycosylations, at two positions: N52 and N78 for the α subunit and N7 and N24 for the β subunit [5]. Structurally, each subunit contains multiple intramolecular disulfide bonds that stabilize its tertiary structure and promote correct protein folding [6]. The α and β subunits associate through non-covalent interactions to form a stable, biologically active heterodimer. The FSHα subunit contains ten conserved cysteine residues forming five disulfide linkages (C7–C31, C10–C60, C28–C82, C32–C84 and C59–C87. The FSHβ subunit contains 12 conserved cysteine residues forming six disulfide linkages (C3–C51, C17–C66, C20–C104, C28–C82, C32–C84 and C87–C94). These disulfide linkages are depicted in Figure 1.

Traditionally, mass spectrometry-based peptide mapping has been employed to elucidate disulfide bond patterns [7]. However, this is a significant challenge with the α subunit. The α subunit contains closely spaced cysteine residues, making these tightly clustered sites highly resistant to efficient cleavage by a wide range of proteases (including trypsin, chymotrypsin, thermolysin, proteinase K, AspN, and elastase). There is no specific and optimal protease capable of selectively cleaving peptides between closely spaced disulfide bonds. Additionally, peptides often contain more than two cysteines, making it difficult to pinpoint exact linkage sites. Methods involving reduction or partial reduction to selectively break disulfide bonds are also difficult to control reliably. Furthermore, there are significant technical difficulties associated with the high degree of glycosylation in Follitropin delta (Rekovelle^®^). Follitropin delta, which was approved in Europe in 2016 [8,9,10], is the first recombinant FSH protein expressed in the human fetal retinal cell-line PER.C6^®^. The PER.C6^®^ cells were genetically engineered to express the human FSH α and β genes and, in addition, the human α 2,3-sialyltransferase (ST3) enzyme to facilitate improved sialylation [11]. The glycosylation level of Follitropin delta is substantially higher than in other rFSH products, such as Follitropin alfa (CHO-derived FSH) and human urinary derived FSH [12].

Given all of the above, accurate disulfide-bond characterization within the α subunit is particularly challenging for this class of protein therapeutics. In contrast, the identification of disulfide bonds in the β subunit is well established and reliably confirmed using LC-MS/MS peptide-mapping approaches involving partial reduction [13].

To address these analytical challenges, we used X-ray crystallography in this study to determine the three-dimensional structure of Follitropin delta. Given the exceptionally high level of glycosylation in the product, the strategy involved forming a complex between rFSH and specific antibody chaperone proteins to facilitate crystallization. By directly visualizing the electron density map associated with disulfide linkages in FSHα within the crystal structure, we provide definitive structural evidence that the disulfide bonds in the recombinant material are consistent with the expected native configuration. This structural validation is intended to support regulatory submissions by demonstrating correct and intact disulfide bonding in the recombinant FSH preparation.

## 2. Results

**Figure 2:**
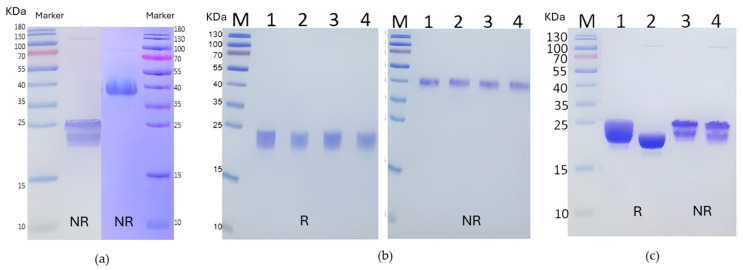
SDS-PAGE analysis of recombinant FSH. (**a**) Non-reducing SDS-PAGE gels of rFSH heated (95 °C, 10 min; left panel) or unheated (right panel) prior to loading. The heated sample show bands (~24–26 kDa) consistent with glycosylated α and β subunits (left), and the non-heated sample shows single ~40 kDa bands, corresponding to the intact FSH heterodimer (right). (**b**) Deglycosylation attempts on rFSH. (1) Untreated, (2) PNGase, (3) Endo H, (4) PNGase F + Endo H. No significant glycan removal was observed by mobility shifts on reducing (left) and non-reducing (right) SDS-PAGE gels. (**c**) Desialidation of rFSH batch. Lanes 1 and 3 are untreated reduced and non-reduced samples, respectively; Lanes 2 and 4 are reduced and non-reduced samples treated with pan-sialidase. rFSH samples were heated (95 °C, 10 min). The clear mobility shift and narrowing of protein bands upon sialidase treatment suggest significant sialic acid removal and improvement in homogeneity of FSH.

As a first step, recombinant human FSH was analyzed by SDS-PAGE under both reducing and non-reducing conditions to assess purity and integrity (Figure 2a). Under reducing conditions, the proteins migrated as two overlapping fuzzy bands corresponding to the glycosylated α and β subunits (~24–26 kDa), as expected for heavily glycosylated proteins. In contrast, non-reducing SDS-PAGE of unheated samples revealed a single band near ~40 kDa, consistent with the intact heterodimer. Heating the samples prior to loading (95 °C, 10 min) caused the heterodimer to dissociate into its component subunits, with mobilities similar to those of the reducing condition (Figure 2a).

To improve crystallization by minimizing glycosylation heterogeneity, we explored enzymatic strategies to simplify the glycans. Initial attempts at enzymatic deglycosylation, using PNGase F and Endo H—individually and in combination—failed to shift the protein’s mobility on SDS-PAGE (Figure 2b). This result suggested that the complex, sialylated glycan structures on mature FSH were resistant to cleavage by these enzymes, consistent with prior reports.

We next employed a bacterial pan-sialidase (Lectenz Bio, San Diego, CA, USA; GE0701) to remove terminal sialic acids. This treatment resulted in a reproducible electrophoretic mobility shift and noticeable band sharpening under both reducing and non-reducing conditions (Figure 2c), indicative of successful sialic acid removal and improved protein homogeneity. These effects were observed regardless of the heating status prior to electrophoresis. Unheated samples maintained the ~40 kDa band, while the heated, sialidase-treated samples dissociated into sharper ~24–26 kDa bands, confirming both sialidase efficacy and the integrity of the heterodimer under native conditions.

Based on these results, desialylated FSH preparations were selected for all subsequent crystallization experiments, either as isolated FSH or in complex with binding partners.

Crystallization trials with desialylated FSH alone yielded clusters of small needle-like crystals in a few conditions. These crystals were not considered suitable for harvesting, their poor quality attributable to the inherent heterogeneity of glycosylation, which can impair crystal lattice formation. They were not pursued further. Instead, a crystallization chaperone strategy was employed, as described below.

### FSH/Anti-FSHα Fab/Anti-Kappa VHH

To aid in formation of diffraction quality crystals, an anti-FSHα Fab fragment was engineered based on the Fv molecule in PDB 1QFW [7], for use in a complex with FSH. Since Fabs are typically flexible at their elbow junction, a well-characterized anti-kappa elbow-binding nanobody (VHH) described in the literature [14], was employed to stabilize the FSH/Fab complex and enhance its crystallizability.

**Figure 3:**
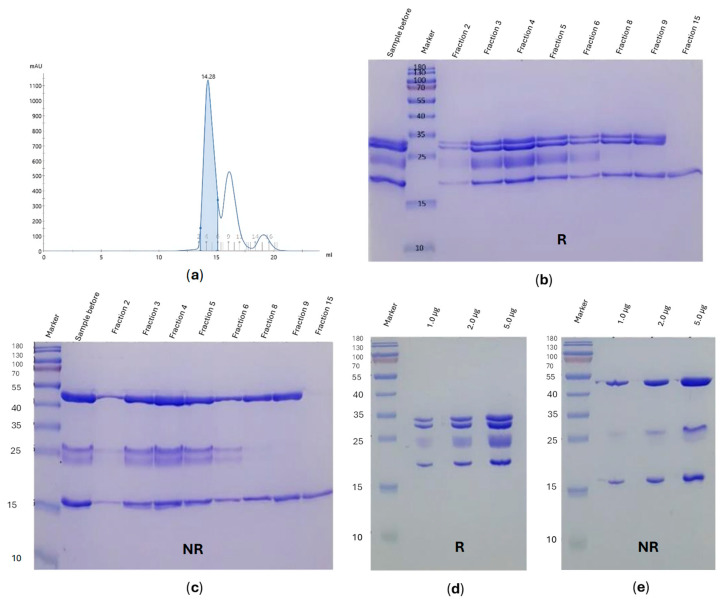
Complex formation of FSH with anti-FSHα Fab and anti-kappa VHH. FSH was incubated with Fab and VHH on ice for two hours, then the well-formed complex purified by SEC (**a**). SDS-PAGE analysis of SEC fractions under reducing (**b**) and non-reducing (**c**) conditions. SDS-PAGE of the final purified complex under reducing (**d**) and non-reducing (**e**) conditions.

FSH formed a stable and well-behaved complex with the anti-FSHα Fab and the anti-kappa VHH, evidenced by a distinct, monodisperse peak on SEC (Figure 3).

Crystallization of the FSH/Fab/VHH ternary complex was carried out using hanging-drop vapor diffusion method at 18 °C. Six crystals were subjected to X-ray diffraction at the Diamond Light Source (BL I03). All six crystals diffracted well, and X-ray data processing indicated that all crystals belonged to the orthorhombic space group P2_1_2_1_2_1_.

One crystal, diffracted to the highest resolution of 2.29 Å, was selected for model refinement (Table 1).

**Figure 4:**
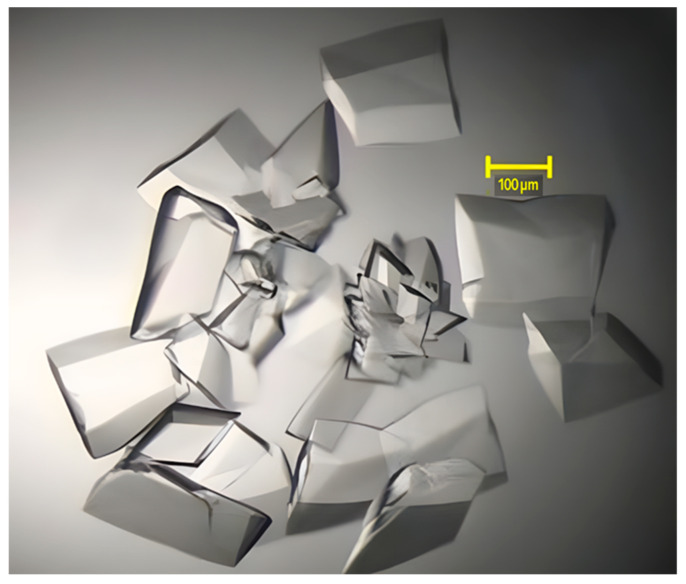
Crystal of desialylated FSH in complex with anti-FSHα Fab and anti-kappa VHH. Hanging drop crystal was performed using 7.5 mg/mL protein, with 3 μL drop sizes and streak seeding from previous crystals, at 18 °C in 0.05 M calcium chloride, 0.1 M Bis-Tris pH 6.5, 30% *v*/*v* PEG 550 MME. The representative image of crystals shown was taken four days after setup.

The fine screen produced crystals with dimensions of ~200 um × 200 um × 50 um (Figure 4).

**Figure 5:**
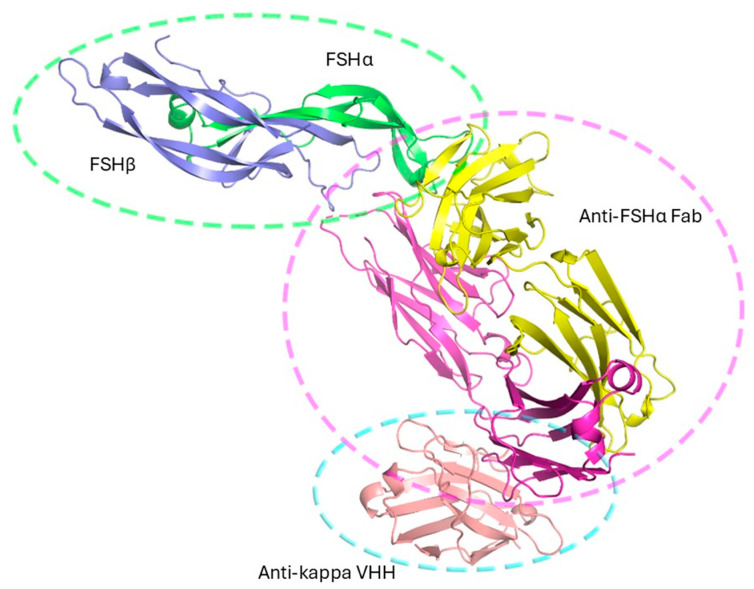
Crystal structure of the FSH/anti-FSHα Fab/anti-kappa VHH complex. The asymmetric unit contains a single copy of the ternary complex comprising human FSH (α-subunit: green; β-subunit: light blue), the anti-FSHα Fab (heavy chain: yellow; light chain: magenta), and an elbow-stabilizing antikappa VHH (salmon).

The crystal structure (PDB: 9YXD) was solved by molecular replacement (Table 1) using Phaser (Phenix suite), with search models for FSH (PDB: 1FL7), Fab (PDB: 7Fab), and VHH (PDB: 8JH7). The asymmetric unit contained one copy of the FSH/Fab/VHH complex (Figure 5), with a solvent content of 66.2%, consistent with a loosely packed but well-ordered lattice.

Model building proceeded iteratively, with manual adjustments guided by interpretable electron density maps. All regions were well resolved except for a flexible loop in the Fab heavy chain (residues S132–G137), and one in the Fab light chain (residues E27-S36) which lacked continuous density.

Crystal packing analysis revealed that each complex interacts with ten symmetry-related neighbors within 4 Å. These contacts include five FSH molecules, three Fabs, and four VHHs, indicating that all components participate in lattice formation. The Fab and VHH, in particular, enhance the number and stability of packing interactions, acting as effective scaffolds that rigidify the complex and facilitate crystallization. All four resolved N-linked glycans—two in FSHβ and one in FSHα—extend into solvent channels and avoid interfering with symmetry contacts. This favorable glycan orientation likely contributed to the high quality of the diffraction data despite the inherent heterogeneity of the glycoprotein.

As shown in Figure 5, FSH adopts the expected architecture observed in previous structures: both α and β subunits form central cystine-knot motifs with three β-hairpin loops, assembling into a compact, elongated heterodimer stabilized by multiple disulfide bonds and polar interactions. The Fab binds to the same α-subunit epitope as its parental Fv (PDB: 1QFW), maintaining specificity. The engineered VHH engages the Fab light chain elbow, consistent with the 8JH7 structure, and was included to rigidify this otherwise flexible region. This design was successful; the complex was well ordered and formed a dense, cooperative lattice mediated by Fab and VHH interfaces.

**Figure 6:**
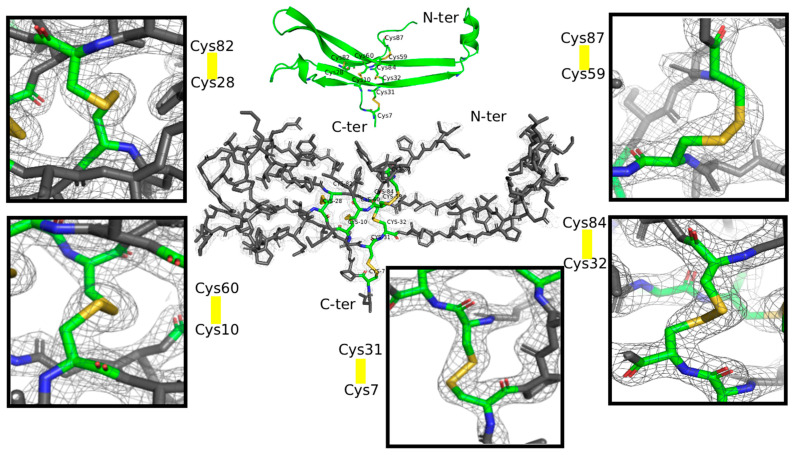
The protein ribbon cartoon is represented in green, highlighting β-strands forming sheets and a single α-helix. Disulfide bonds (yellow) between cysteine residues (C7–C31, C10–C60, C28-C82, C32–C84, and C59–C87) are shown as sticks, with labeled cysteine positions ensuring structural stability of the glycoprotein hormone subunit. The 2.29 Å 2Fo–Fc electron density (in gray) shown contoured at 1.0 σ around the Follitropin delta α-subunit and a zoom in on each disulfide bond allowed unambiguous assignment of canonical and intact S–S connectivity in this subunit.

At 2.29 Å resolution, the electron density was of sufficient quality to definitively assign all disulfide bonds in the FSH α and β subunits (Figure 6). Each cysteine pairing was supported by continuous and unambiguous 2Fo–Fc density, clearly demonstrating that crystals of Follitropin delta have a disulfide architecture that is fully consistent with that observed in previously determined structures of FSH from other biological sources (see Section 3 below).

All structural elements were well resolved except for a flexible loop in the Fab light chain (D30-Y21), which lacked continuous electron density. Extra density corresponding to all four N-linked glycans—two on FSHα and two on FSHβ—were observed at this resolution.

## 3. Discussion

Accurate disulfide mapping is a critical attribute for recombinant FSH products, as incorrect disulfide pairing can lead to misfolded or inactive protein, potentially affecting efficacy or immunogenicity [15]. Traditionally, mass spectrometry-based peptide mapping has been employed to elucidate disulfide bond patterns [16]. However, in our case, attempts at disulfide mapping using the isolated recombinant FSHα subunit were ultimately unsuccessful due to a combination of factors: the high number of cysteines, the resulting disulfide bonds in close proximity to each other and to glycosylated residues, susceptibility to disulfide scrambling during sample preparation, and incomplete peptide fragmentation under non-reducing conditions, and the complexity of glycosylation conferred by the host PER.C6^®^ cells. Despite extensive efforts involving multiple rounds of proteolytic digestion, deglycosylation, and partial reduction strategies, the heavily glycosylated and compact nature of FSHα prevented confident disulfide bond assignment. These negative results led us to pursue X-ray crystallography as a definitive approach to resolve the disulfide topology of recombinant FSH under native conditions.

Literature offers several structural precedents for glycoprotein hormones, with most relying on deglycosylation, desialylation, or protein engineering to improve lattice formation.

Only one structure of native-like FSH in isolation has been reported (PDB: 1FL7); the reported hormone was produced in *Trichoplusia ni* (Hi5), insect cells and engineered to remove a β-subunit glycosylation site (T26A) to reduce heterogeneity and promote crystallization [17]. Crystal structures of FSH in complex with the extracellular domain of the FSH receptor (FSHR) have been reported: 1XWD (2.92 Å), 4AY9 (2.50 Å), and 4MQW (2.90 Å). In these reported studies, FSH was co-expressed with the FSHR ectodomain, and hormone-receptor interfaces contributed to lattice stabilization. While these structures establish the canonical disulfide architecture of both α and β subunits, the strategies used to obtain them were not compatible with our objective—structural validation of disulfide connectivity in the clinical recombinant FSH product, without genetic modification to reduce glycosylation or co-expression with the receptor ecto domain (our attempts to express the ecto domain on its own failed.

Chemical and enzymatic strategies have been pursued with human chorionic gonadotropin (hCG), which shares the FSHα subunit and is similarly glycosylated. Early crystal structures of hCG (1HCN, 1HRP) were obtained after chemical deglycosylation with anhydrous hydrogen fluoride (HF) [18,19], a harsh treatment that can damage the protein backbone. Lustbader et al. [20] demonstrated that enzymatic desialylation using neuraminidase led to better crystal quality and reduced peptide bond cleavage compared to HF-treated samples. Their findings suggested that negatively charged terminal sialic acids, rather than total glycan content, are the principal inhibitors of lattice formation. Later, a ternary complex of fully glycosylated hCG and two Fv fragments was crystallized, which resulted in a structure, albeit at lower resolution (1QFW), highlighting the utility of crystallization chaperones in overcoming glycan-related barriers.

Taken together, these precedents motivated a crystallization strategy incorporating desialylation and engineered antibody fragments as auxiliary partners to shield glycan-rich regions, stabilize the complex, and promote consistent packing, thereby enabling structure determination of native recombinant FSH. Recognizing the potential for high attrition due to glycan heterogeneity, structural flexibility, and diffraction variability, multiple crystallization approaches were pursued for a ternary FSH/Fab/VHH complex, yielding high-quality crystals and structure at 2.29 Å.

The structure obtained revealed a single complex per asymmetric unit and extensive lattice interactions mediated predominantly by the Fab and VHH, which stabilized the architecture and enabled nucleation and crystal growth. Glycans projected into solvent channels, avoiding steric interference. Critically, the resulting electron density maps permitted unambiguous assignment of all intramolecular disulfide bonds within the intact FSH heterodimer, thereby directly establishing the cysteine connectivity of crystallized recombinant Follitropin delta at structural resolution.

As with all macromolecular crystallographic analyses, these conclusions pertain to the population captured in the crystalline lattice and should be interpreted within that context. However, concerns about crystallization selectivity must be evaluated in light of whether alternative molecular species are present at meaningful levels in the starting material. The protein used for crystallization was prepared to high purity (97%) using procedures representative of the therapeutic manufacturing process. Under standard quality control parameters for recombinant therapeutics, this purity level indicates a dominant, well-formed molecular species with minimal heterogeneous subpopulations.

While crystallization can theoretically select for particular conformers when multiple species coexist, this selectivity becomes moot in the absence of substantial heterogeneity. The high purity of the crystallized material supports the interpretation that the observed disulfide connectivity reflects the intended and clinically relevant molecular species rather than a minor, selectively enriched variant. If alternative disulfide connectivities were present at appreciable levels, they would be expected to interfere with crystallization or manifest as interpretable signals within the crystal structure itself.

Importantly, the use of Fab and VHH crystallization chaperones further reduces the likelihood of selective exclusion based on disulfide heterogeneity. These chaperones mediate extensive crystal contacts but do not directly interact with the cysteine-containing regions of FSH. Alternative disulfide connectivities, if present at significant levels, would be expected to alter local structure and manifest as disorder, multiple conformations, or discontinuous electron density in cysteine-containing regions. Instead, all disulfide bonds are supported by continuous and unambiguous electron density, permitting confident assignment of cysteine pairing across all five disulfide bonds in the FSHα subunit.

Protein crystals routinely accommodate microheterogeneity arising from flexible regions, partial disorder, and alternate conformations. The successful crystallization and high-resolution structure determination of the FSH/Fab/VHH ternary complex therefore indicates both the dominance of a single well-ordered species in solution and the structural compatibility of that species with lattice formation. While crystallography cannot exclude the presence of rare alternative disulfide isomers below the detection threshold, the combination of high starting purity, absence of structural disorder in cysteine-containing regions, and unambiguous electron density provides strong evidence that the canonical disulfide architecture is correctly and predominantly formed in recombinant Follitropin delta.

The crystallographic analysis presented here therefore serves as a robust and orthogonal validation of cysteine connectivity, complementing analytical methods that are challenged by clustered cysteine motifs. The structure provides a reference standard for quality control, offering a direct method for visualizing disulfide linkages within the context of the intact, folded protein precisely the form relevant to therapeutic function.

## 4. Materials and Methods

### 4.1. Protein Production

Recombinant FSH: A batch of recombinant human FSH, produced in PER.C6^®^ cells at Bio-Technology General (Kiryat Malachi, Israel) Ltd., was used in this study. The sample was stored and transferred at −20 °C degrees, in a formulation buffer of 1 mM Na2HPO4·12H2O with 0.5 mg/mL L-Methionine and 0.005 mg/mL Polysorbate 20, pH 6.75 ± 0.2, at 0.63 mg/mL concentration.

Due to the difficulty in removing FSH glycans with PNGase F, a pan-sialidase (Lectenz Bio, San Diego, CA, USA, catalog No. GE0701) was used to enzymatically remove terminal sialic acid residues. This step served to reduce heterogeneity and the overall negative surface charge, thereby improving crystallizability. One 5000-unit vial of sialidase was used per 10 mg of FSH, and digestion was monitored by reducing and non-reducing SDS-PAGE. The desialylated protein was concentrated to 5.6 mg/mL in 20 mM HEPES pH 7.5, 100 mM NaCl and used directly for complex formation.

Anti-FSHα Fab: As a strategy to aid crystallization, an anti-FSHα Fab fragment was engineered based on the sequence of the Fv structure in PDB [21] 1QFW [7]. An Ala12Ser mutation (mature light chain numbering; Table 2) was introduced into the Fab light chain construct to correct the only mismatch in the binding interface for the elbow-stabilizing anti-kappa VHH nanobody, based on PDB structure 8JH7 [22], which we additionally employed. This design ensured co-binding of the VHH to enhance lattice formation during crystallization.

Codon-optimized sequences of the Fab heavy and light chains were synthesized and cloned into pTT5 mammalian expression vectors. The constructs were co-transfected into Expi293F cells (1 L scale) and cultured at 37 °C. The supernatant was harvested 120 h post-transfection.

The secreted Fab was purified by affinity chromatography using a CaptureSelect IgG-CH1 column. After washing with PBS, the Fab was eluted with 20 mM acetate pH 3.5, 150 mM NaCl, and immediately neutralized with 1 M Tris pH 8.0. The eluate was then purified by size exclusion column (SEC) on a Superdex 200 16/600 column equilibrated in 50 mM Tris pH 7.5, 150 mM NaCl. The purified Fab was concentrated and flash-frozen in liquid nitrogen for storage. Electrospray ionization mass spectrometry (ESI-MS) was used to confirm protein purity and consistency with the expected disulfide bond formation.

Anti-kappa VHH: To support Fab elbow stabilization and aid crystallization of the FSH/Fab complex, an anti-kappa nanobody (VHH) was produced. This single-domain VHH, derived from PDB structure 8JH7 [22], specifically recognizes the constant region of the human kappa light chain.

A codon-optimized sequence of the anti-kappa VHH was cloned into a pET26b vector containing a pelB signal sequence and transformed into *E. coli* BL21(DE3) cells. A 2 L LB culture was grown at 37 °C to an OD600 of ~0.6 and induced with 0.1 mM IPTG. Expression continued at 16 °C for 48 h.

Cells were harvested by centrifugation and resuspended in lysis buffer containing 20 mM HEPES pH 7.5, 100 mM NaCl, 10 mM imidazole, 5 mM MgCl_2_, 1 mM PMSF, 50 μg/mL DNase I, 100 μg/mL lysozyme, and protease inhibitor tablets (1 per 50 mL). Lysis was performed by sonication, and the clarified lysate was applied to a Ni affinity column pre-equilibrated with Buffer A (20 mM HEPES pH 7.5, 500 mM NaCl, 40 mM imidazole). The column was washed extensively with Buffer A, and the bound VHH was eluted with Buffer B (20 mM HEPES pH 7.5, 100 mM NaCl, 500 mM imidazole).

The eluate was concentrated and further purified by SEC on a Superdex 75 16/600 column equilibrated in 10 mM HEPES pH 7.5, 100 mM NaCl, 10% glycerol. Peak fractions were pooled, concentrated, and flash-frozen in liquid nitrogen for storage. ESI-MS was used to confirm protein purity and consistency with the expected disulfide bond formation.

### 4.2. Crystallization

FSH/Fab/VHH complex: A ternary complex was prepared by incubating 1.5 mg desialylated FSH with 4.83 mg Fab and 1.9 mg VHH on ice for two hours, followed by SEC purification as above. Peak fractions were pooled and concentrated to 7.3 mg/mL and used for crystallization screening.

Crystallization screening of this preparation was performed using the sitting-drop vapor diffusion method in a 96-well format at 18 °C. Each drop consisted of 150 nL protein solution mixed with 150 nL crystallant solution. Harvestable crystals were cryoprotected as needed and flash-cooled in liquid nitrogen and screened for X-ray diffraction.

Fine-screen conditions were explored around the original hit conditions that yielded well-diffracting crystals, with crystallization trials set up using the hanging-drop vapor diffusion method at 18 °C. Drops consisting of protein solution and crystallant solution were mixed at three different ratios: 1 μL + 2 μL, 1.5 μL + 1.5 μL, and 2 μL + 1 μL. Larger drop sizes were used to encourage growth of bigger, better diffracting crystals. Streak seeding with crystals from initial hits was applied to all drops to enhance nucleation and improve crystal quality. The optimized crystallization conditions were intrinsically cryoprotective and required no additional cryoprotectant. These conditions yielded well-diffracting crystals of the ternary FSH/Fab/VHH complex.

### 4.3. X-Ray Diffraction

Crystals were screened and complete diffraction datasets were collected at 100 K with a wavelength of 0.97625 Å from well-diffracting crystals of the FSH/Fab/VHH complex at the Diamond Light Source (DLS beam line I03, Oxfordshire, UK).

### 4.4. Structure Determination

Diffraction images were processed automatically at the synchrotron beamlines using the xia2-dials 3dii pipeline [23,24]. Merged and scaled intensities were obtained using AIMLESS [25].

Initial phases were obtained by molecular replacement using Phaser [26] (within the Phenix suite), with search models including FSH (PDB: 1FL7 [17]), Fab (PDB: 7Fab [27]), and VHH (from PDB: 8JH7 [22]). The model was manually built into interpretable electron density using Coot, with iterative cycles of model building and refinement carried out in Phenix [28] and Coot [29].

Particular attention was given to disulfide bond connectivity. Electron density corresponding to cysteine residues was carefully inspected to confirm the presence and correct pairing of disulfide linkages. The final model was validated using MolProbity 4-5-2 [30].

## Figures and Tables

**Table 1:** Data collection, structure determination and refinement statistics for FSH complex with anti-FSHα Fab and anti-kappa VHH. Data in parenthesis correspond to the highest resolution shell.

Data Collection & Refinement Statistics	rFSH
Beamline	DLS I03
Wavelength (Å)	0.97625
Resolution range (Å)	29.85–2.29 (2.35–2.29)
Space group	P2_1_2_1_2_1_
Unit cell parameters (Å; °)	a = 76.01, b = 101.10, c = 161.24; α = β = γ = 90
Matthew coefficient (Å3/Da)	3.65
Molecules per asymmetric unit	1
Total reflections	739,731 (40,781)
Unique reflections	56,196 (3969)
Multiplicity	13.2 (10.3)
Completeness (%)	98.6 (86.8)
Mean I/sigma(I)	33.8 (5.5)
Wilson B-factor	50.15
R-merge/R-meas/Rpim	0.044 (0.29)/0.045 (0.305)/0.012 (0.093)
CC1/2	1 (0.966)
Reflections used in refinement	55,871 (2341)
Reflections used for R-free	2814 (131)
Rwork/Rfree	0.1947 (0.2284)
Total no. of non-hydrogen atoms (protein)	6254
No. of protein/solvent residues	743/454
RMSD bond lengths, bond angles (Å; °)	0.007/0.95
Ramachandran favored/allowed/outliers/rotamer outliers (%)	97.13/2.74/0.14/2.33
Clashscore	8.58
Average B-factor/protein/ligands/solvent	52.10/51.49/72.97/55.59

**Table 2:** Expressed protein sequences of crystallization chaperone proteins. Signal peptide sequences are underlined.

Construct	Sequence
Anti-FSHα Fab LC	MKHLWFFLLLVAAPRWVLSDIELTQSPDSLSVSLGQRATISCRASESVDSYGNSFMQWY QQKPGQPKLLIYRASNLEGSIPARFSGTGSRTDFTLTINPVEADDVATYYCQQSDEYPYM YTFGGGTKLEIKRTVAAPSVFIFPPSDEQLKSGTASVVCLLNNFYPREAKVQWKVDNALQSGN SQESVTEQDSKDSTYSLSSTLTLSKADYEKHKVYACEVTHQGLSSPVTKSFNRGEC
Anti-FSHα Fab HC	MVLQTQVFISLLWISGAYGQVQLQQSGAELVKPGASVKLSCKASDYTFTSYWMHWVKQRP GQGLEWIGEINPTNGRTYYNEKFKSKATLTDVKSSSTAYMQLSSLTSEDSAVTTCATTYGNSFDY WGQGTTVTVSSASTKGPSVFPLAPSSKSTSGGTAALGCLVKDYFPEPVTVSWNSGALTSVHTFPA VLQSSGLYSLSSVVTVPSSSLGTQTYICNVNHKPSTNTKVDKKVEPKSCDKTHT
Anti-kappa VHH	MKYLLPTAAAGLLLLAAQPAMAHHHHHHQVQLQESGGGLVQPGGSLRLSCAASGRTISRYA MSWFRQAPGKEREFVAVARRSGDGAFYADSVQGRFTVSRDDAKNTVYLQMNSLKPEDTAYYCA IDSDTFYSGSYDYGWGQGTQVTVSS

## Data Availability

The original data presented in the study are openly available in https://www.rcsb.org/structure/unreleased/9YXD (accessed on 19 February 2026) Crystallographic coordinates and structure factors have been deposited to the Protein Data Bank (www.rcsb.org (accessed on 19 February 2026)) as PDB ID 9YXD.

## Abbreviations

The following abbreviations are used in this manuscript:

cAMP	Cyclic adenosine-monophosphate
CG	Choriogonadotropin
CHO	Chinese hamster ovary
ESI-MS	Electrospray ionization mass spectrometry
FSHR	FSH receptor
GC	Granulosa cells
GnRH	Gonadotropin-releasing hormone
HF	Hydrogen fluoride
LH	Luteinizing hormone
rFSH	Recombinant follicle stimulating hormone
SEC	Size exclusion column
ST3	α2,3-sialyltransferase
VHH	Anti-kappa nanobody
